# Mesenchymal stromal cells highly expressing Sca-1 promote breast cancer lung metastasis through recruiting myeloid cells

**DOI:** 10.1038/s41419-025-07845-0

**Published:** 2025-07-09

**Authors:** Lijuan Cao, Yanan Li, Minghui Ou, Artem Smirnov, Rui Liu, Tingting Wang, Xiao Su, Peishan Li, Mancini Mara, Eleonora Candi, Zhiyuan Zheng, Gerry Melino, Changshun Shao, Yufang Shi

**Affiliations:** 1 The Third Affiliated Hospital of Soochow University, Institutes for Translational Medicine, State Key Laboratory of Radiation Medicine and Protection, Suzhou Medical College of Soochow University, Suzhou, Jiangsu China; 2 Department of Experimental Medicine, TOR, University of Rome Tor Vergata, Rome, Italy; 3 IDI-IRCCS, Rome, Italy; 4 State Key Laboratory of Macromolecular Drugs and Large-scale Preparation, School of 12 Pharmaceutical Sciences, Wenzhou Medical University, Wenzhou, Zhejiang China

**Keywords:** Mesenchymal stem cells, Cancer microenvironment

## Abstract

Mesenchymal stem/stromal cells (MSCs) are integral components of the tumor microenvironment and critical for the colonization of disseminated cancer cells; specifically, stem cell antigen (Sca-1) is recognized as a surface marker of MSCs. In this study, we found that MSCs highly expressing Sca-1 are positively associated with lung metastasis. MSCs derived from the lungs of mice bearing metastasized breast tumors (LMSCs) exhibited higher level of Sca-1 compared to those with adenoma. When co-injected with 4T1 cells intravenously, Sca-1^high^ LMSCs resulted in more tumor nodules in lung tissue than Sca-1^low^ LMSCs. Furthermore, Sca-1^high^ LMSCs expressed higher levels of CCL2, CCL7, and CXCL1 than Sca-1^low^ LMSCs. Sca-1^high^ LMSCs can directly recruit 4T1 cells through producing CXCL1. Additionally, Sca-1^high^ LMSCs are highly potent in recruiting immune cells of the myeloid lineage (neutrophils and macrophages) to the lungs. Inhibition of macrophage chemotaxis by Bindarit, an inhibitor of CCL2/7/8 transcription, decreased the lung tumor burden induced by Sca-1^high^ MSCs. Using *Ccr5*^−/−^ mice, it was further confirmed that Sca-1^high^ LMSCs promote tumorigenesis by recruiting macrophages, further supporting that the increased recruitment of macrophages mediates the pro-metastasis effect of Sca-1^high^ LMSCs. Collectively, this study demonstrated that Sca-1^high^ LMSCs and their effectors could be targeted to inhibit breast cancer metastasis to the lung.

## Introduction

Mesenchymal cells, derived from mesoderm or ectoderm, are primarily recognized for their roles in providing structural support to organs, synthesizing and remodeling the extracellular matrix (ECM), and regulating development, homeostasis, and tissue repair. Mesenchymal stem cells (MSCs), also known as mesenchymal stromal cells, exist in many tissues and are known to actively migrate to site of tissue injury, where they participate in wound repair [1, 2]. As stem cells, MSCs possess the potential for multi-directional differentiation, including adipogenesis, osteogenesis, and chondrogenesis. Additionally, their immunoregulatory function has gained recognition in recent years. MSCs licensed by IFN-γ, plus any one of TNF-α, IL-1α, or IL-1β in the inflammatory environment, acquire the immunosuppressive function. Consequently, they upregulate chemokines and effectors to modulate immune cells-induced inflammation response and promote tissue repair [3, 4].

Stem cell antigen (Sca-1) expression is mainly on mesoderm-derived cells, although it is not restricted to the stem cell/progenitor cell population. Several surface markers have been utilized to identify mouse MSCs, with Sca-1 being one of the most common. Sca-1 and Sca-2 are members of Ly-6 family of interferon-inducible lymphocyte activation proteins, encoded by genes located on mouse chromosome 15. Sca-1 is an 18 kDa mouse glycosyphosphatidylinositol (GPI)-linked cell surface protein and is also known as Ly6A/E encodes by the strain-specific Ly6a/e gene. When present on the cell membrane, Sca-1 is associated with protein tyrosine kinases and lipid rafts, suggesting its potential involvement in signal transduction. Although the syntenic region of the Sca-1 gene is found on human chromosome 8, it does not seem to encode a functional equivalent of the mouse Sca-1. The human counterpart remains elusive. Interestingly, a population of Sca-1^+^-like cells was isolated from the adult human heart with an anti-mouse Sca-1 antibody [5], indicating a human ortholog or related protein may exist in humans.

Tumors are often regarded as “wounds that never heal”, with metastatic dissemination representing the final stage of a deteriorating process. These ectopic sites, known as metastatic niches, provide activated harbors for tumor cells, driving their colonization and proliferation. In recent years, evidence has emerged suggesting that primary tumors prime certain cells for tumor cell engraftment, with bone marrow-derived cell clusters (BMDCs) in the ‘pre-metastatic microenvironment’ creating a conducive environment for tumor cell adhesion and invasion. The pre-metastatic microenvironment, also termed the ‘pre-metastatic niche’ (PMNs), refers to microenvironments in distant organs that are capable of supporting the survival and outgrowth of tumor cells before their arrival at these sites. BMDCs play vital role in the formation of pre-metastatic niche. Macrophages, as key determinants for the formation of PMNs, are mobilized to the bloodstream and subsequently clustered in pre-metastatic sites by various tumor-secreted factors, such as CCL2, CFS-1, VEGF, PLGF, TNF-α, TGF-β, tissue inhibitor of metallopeptidase-1 (TIMP-1), and exosomes [6].

We previously reported that LMSCs play a critical role in establishing PMNs and metastatic niches for breast cancer cells [7]. In this study, we demonstrate that along with the development of breast cancer, Sca-1 expression is increased in LMSCs. Sca-1^high^ LMSCs could effectively promote the colonization of tumor cells in the lung. Sca-1^high^ LMSCs can directly increase tumor cell migration through the secretion of chemokines such as CXCL1. Furthermore, the presence of macrophages increases in tumors following the co-injection of 4T1 and Sca-1^high^ LMSCs compared to 4T1 and Sca-1^low^ LMSCs. Inhibiting macrophage infiltration in mice co-injected with 4T1 and Sca-1^high^ LMSCs reduces tumor nodules to corresponding control level. Taken together, Sca-1^high^ LMSCs promote tumor metastasis by recruiting tumor cells and increasing macrophage infiltration through the secretion of chemokines.

## Results

### Tumor-associated LMSCs highly express Sca-1

**Fig. 1:**
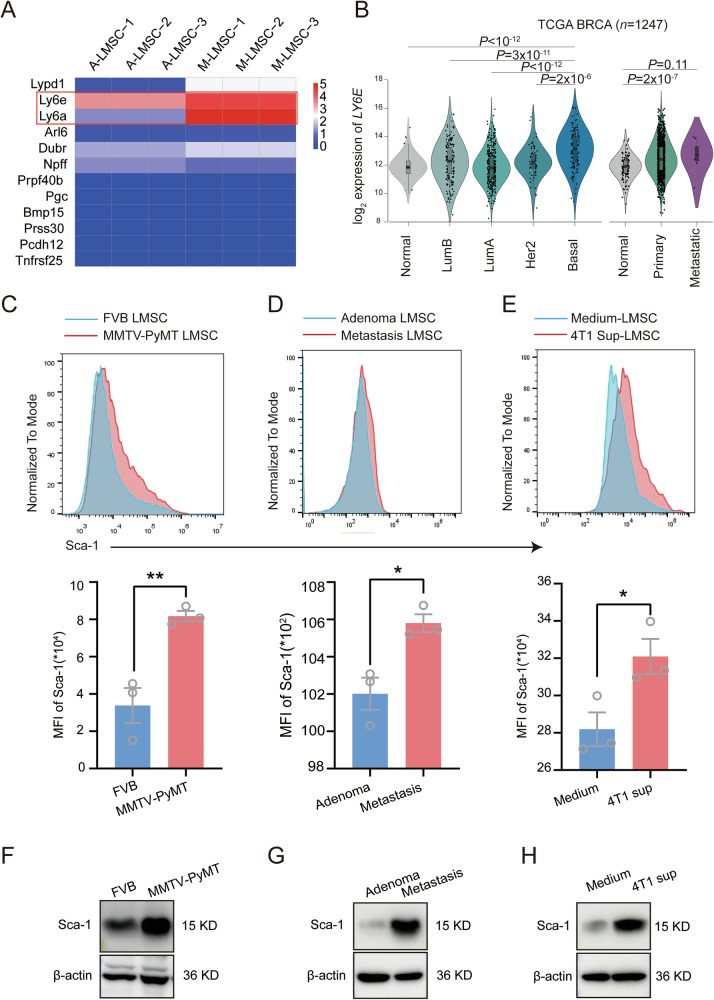
Tumor associated LMSCs show higher expression of Sca-1. **A** RNA sequencing analysis of LMSCs derived from MMTV-PyMT mice at adenomatous and metastatic stages (*n* = 3). **B** Viloin plots showing LY6E expression in TCGA BRCA samples (*n* = 1247) based on subtype (left) or tumor type (right). Single values are shown as dots. **C** Comparison of Sca-1 expression level in LMSCs derived from FVB mice and MMTV-PyMT mice, respectively (*n* = 3). **D** Comparison of Sca-1 expression level in LMSCs derived from MMTV-PyMT mice at adenoma and metastatic stages (*n* = 3). **E** Comparison of Sca-1 expression level in LMSCs cultured with normal medium and 4T1 cells-derived supernatant, respectively (*n* = 3). **F** Lung mesenchymal stem cells (LMSCs) derived from FVB or MMTV-PyMT mice were collected for immunoblot analysis. **G** Western blot analysis of LMSCs derived from adenoma and metastatic MMTV mice. **H** Lung mesenchymal stem cells treated with or without 4T1 supernatant were analyzed by Western blot. Data are mean ± SEM of biologically independent samples. Statistical analysis was determined by one-tailed unpaired *t* test (**C**–**E**) or one-way ANOVA (**B**). *p < 0.05, **p < 0.01.

In our previous study, we discovered the involvement of LMSCs in the establishment of metastatic niches for breast cancer cells [7]. By analyzing RNA-seq datasets generated previously (GSE125591), we found numerous differentially expressed genes in LMSCs derived from MMTV-PyMT mice at different stages of tumor development. Notably, *Sca-1* (*Ly6a/e*), a hallmark of MSCs, showed elevated expression in LMSCs from the metastasis stage compared to the adenoma stage (Fig. 1A). Furthermore, analysis of the TCGA BRCA breast cancer cohort revealed a significantly higher expression level of *LY6E* in breast tumor patients compared to healthy individuals, especially in basal subtype of BRCA. Moreover, metastatic samples showed a trend towards an increased expression of *LY6E*, although the differences did not reach statistical significance (Fig. 1B). To corroborate these findings, we measured the Sca-1 protein level in wild-type (WT) LMSCs and MMTV-PyMT LMSCS using flow cytometry. Consistently, MMTV-PyMT LMSCs exhibited higher fluorescence intensity, indicating elevated Sca-1 expression (Fig. 1C). Additionally, we assessed Sca-1 expression levels in LMSCs derived from different metastatic stages, finding that LMSCs from metastatic stages displayed higher fluorescence intensity compared to those from the adenoma stage (Fig. 1D). These observations suggest that Sca-1 may define a subset of tumor-associated MSCs that promote metastasis. To investigate whether the altered Sca-1 expression is influenced by tumor cells, we cultured wild-type (WT) LSMCs with conditioned medium containing 4T1 cell culture supernatant. As expected, the 4T1-conditioned medium significantly increased Sca-1 expression on LMSCs (Fig. 1E). Western blotting analysis further confirmed the elevated expression of Sca-1 in tumor-associated LMSCs. Taken together, these findings indicate that breast cancer-associated LMSCs are heterogeneous, and Sca-1 expression is associated with the emergence of a subset of pro-metastatic LMSCs.

### Sca-1^high^ LMSCs promote breast cancer lung metastasis

**Fig. 2:**
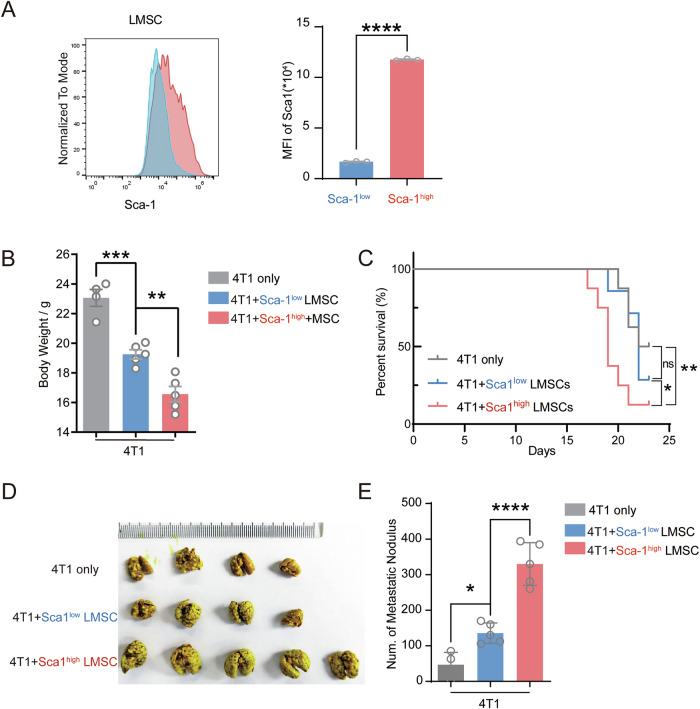
Sca-1^high^ LMSCs are more potent in promoting 4T1 tumor cell colonization in lung. **A** Identification of sorted Sca-1^low^ and Sca-1^high^ LMSCs (*n* = 3). **B**, **C** Body weights (*n* = 4 or 5) and survival rate (*n* = 7 or 8) of mice 2 weeks after co-injection of 4T1 tumor cells and Sca-1^low^ or Sca-1^high^ LMSCs, respectively. **D**, **E** Nodules of 4T1 colonization in lungs were stained by Bouin’s solution (*n* = 4 or 5). Data are mean ± SEM of biologically independent samples. Statistical analysis was determined by one-tailed unpaired *t* test (**A**) and one-way ANOVA with Bonferroni’s multiple comparisons test (**B**, **D**). *p < 0.05, **p < 0.01, ***p < 0.001.

While we observed differential Sca-1 expression in LMSCs, it remained unclear whether this expression level was associated with metastasis-promoting properties. To address this, we enriched Sca-1^low^ LMSCs and Sca-1^high^ LMSCs using magnetic-activated cell sorting (MACS) (Fig. 2A). To assess the impact of Sca-1 expression levels on tumor metastasis, we established a mouse model of breast cancer lung colonization. In this model, 4T1 cells were co-injected with either Sca-1^low^ or Sca-1^high^ LMSCs into Balb/c mice via intravenous injection. Mice injected with 4T1 and Sca-1^low^ LMSCs experienced weight loss, while those injected with 4T1 mixed with Sca-1^high^ LMSCs exhibited significant weight loss, ~33% greater than the 4T1-only group (Fig. 2B). Weight loss is closely associated with cancer progression and is a common side effect experienced by cancer patients [8]. More importantly, mice injected with 4T1 admixed with Sca-1^high^ LMSCs exhibited shorter survival than those receiving 4T1 alone or 4T1 admixed with Sca-1^low^ LMSCs (Fig. 2C). Additionally, we quantified lung metastatic nodule formation. Consistently, Sca-1^low^ LMSCs promoted the formation of lung metastatic nodules compared to the 4T1-only group (Fig. 2D). Intriguingly, Sca-1^high^ LMSCs led to even larger metastatic nodules than Sca-1^low^ LMSCs (Fig. 2D). Moreover, the number of lung nodules was also quantified, revealing that co-administration of 4T1 cells with Sca-1^high^ LMSCs resulted in a greater number of lung nodules compared to the other groups (Fig. 2E). In conclusion, our findings suggest that Sca-1 expression facilitates breast cancer lung metastasis, with Sca-1^high^ LMSCs exhibiting a stronger capacity to support lung metastasis than Sca-1^low^ LMSCs.

### Sca-1^high^ LMSCs recruit tumor cells via CXCL1

**Fig. 3:**
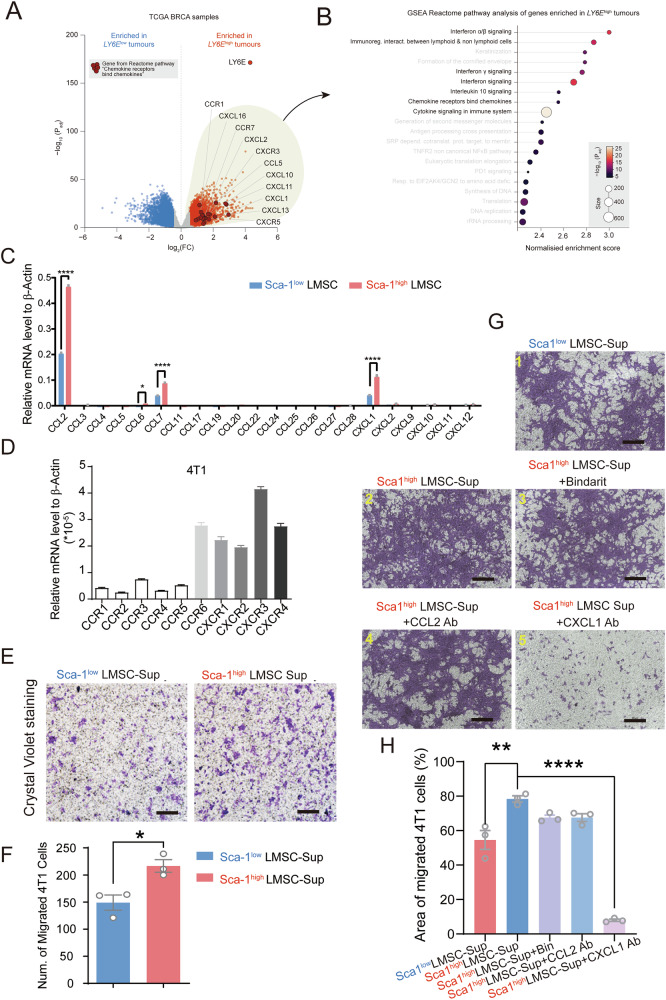
Sca-1^high^ LMSCs promote 4T1 cell migration through CXCL1. **A** Volcano plot showing genes enriched in *LY6E*^high^ or *LY6E*^low^ samples from TCGA BRCA cohort. Genes from Reactome pathway “*Chemokine receptors bind chemokines*” are highlighted. **B** GSEA Reactome enrichment analysis of genes enriched in *LY6E*^high^ samples from (**A**). Inflammation-related pathways are in black, and other pathways are in gray. **C** Expression levels of different genes associated with cytokines in Sca-1^low^ and Sca-1^high^ LMSCs were determined by real-time PCR (*n* = 3). **D** Expression levels of chemokine receptors on the 4T1 cells were also detected by real-time PCR (*n* = 3). **E**, **F** Transferred 4T1 cells cultured with Sca-1^low^ and Sca-1^high^ LMSCs supernatant separately were analyzed by crystal violet staining (*n* = 3). Scale bar = 500 μm. **G**, **H** Sca-1^high^ LMSCs supernatant was treated with Bindarit, CCL2 antibody, or CXCL1 antibody, respectively, and then employed to culture 4T1 cells (*n* = 3). The migrated 4T1 cells were stained by crystal violet. Scale bar = 500 μm. Data are mean ± SEM of biologically independent samples. Statistical analysis was determined by one-tailed unpaired *t* test (**A**, **D**) and one-way ANOVA with Bonferroni’s multiple comparisons test (**F**). *p < 0.05, ****p < 0.0001.

To understand a possible association between Sca-1 and TME, we performed differential expression analysis between two cohorts of breast cancer samples (TCGA) with high or low levels of *LY6E* (Fig. 3A). The genes enriched in *LY6E*^high^ tumors were strongly and significantly associated with chemokine signaling (Fig. 3B), consistent with previous reports [9]. We aimed to investigate whether Sca-1 modulates the expression of key cytokines to promote tumor metastasis. To address this, we conducted gene expression profiling of Sca-1^low^ and Sca-1^high^ LMSCs. Interestingly, our chemokine qPCR-array revealed significantly elevated levels of CCL2, CCL7, and CXCL1 in Sca-1^low^ LMSCs (Fig. 3C). Furthermore, these cytokines were further upregulated in Sca-1^high^ LMSCs (Fig. 3C). To further explore whether LMSCs could recruit 4T1 cells to the lungs via secreted cytokines, we examined the expression of their receptors on the 4T1 cell line. Our results showed low but detectable levels of CCR2, CCR3, and CXCR2, which are the classical receptors for CCL2 (CCR2), CCL7 (CCR1, CCR2, CCR3), and CXCL1 (CXCR2), respectively (Fig. 3D). Subsequently, we utilized a trans-well system to co-cultured the 4T1 cell line with supernatant from Sca-1^low^ or Sca-1^high^ LMSCs, respectively. Surprisingly, crystal violet staining results suggested that 4T1 cells migrated to the lower chamber, with significantly more 4T1 cells observed in the Sca-1^high^ LMSCs treated group (Fig. 3E-F). To ascertain which factor mediates the recruitment of 4T1 cells, we added Bindarit (a selective inhibitor of the monocyte chemotactic proteins CCL2, CCL7, and CCL8 by inhibiting gene transcription), CCL2 antibody, and CXCL1 antibody into the 4T1 culture medium separately. We found a notable reduction in 4T1 cells migration when Sca-1^high^ LMSCs supernatant was supplemented with the CXCL1 antibody (Fig. 3G, H). Taken together, these findings demonstrate that Sca-1^high^ LMSCs can recruit 4T1 cells directly through the chemokine-receptor interaction, specifically via CXCL1.

### Sca-1^high^ LMSCs promote infiltration of myeloid immune cells in metastatic lung tissue

**Fig. 4:**
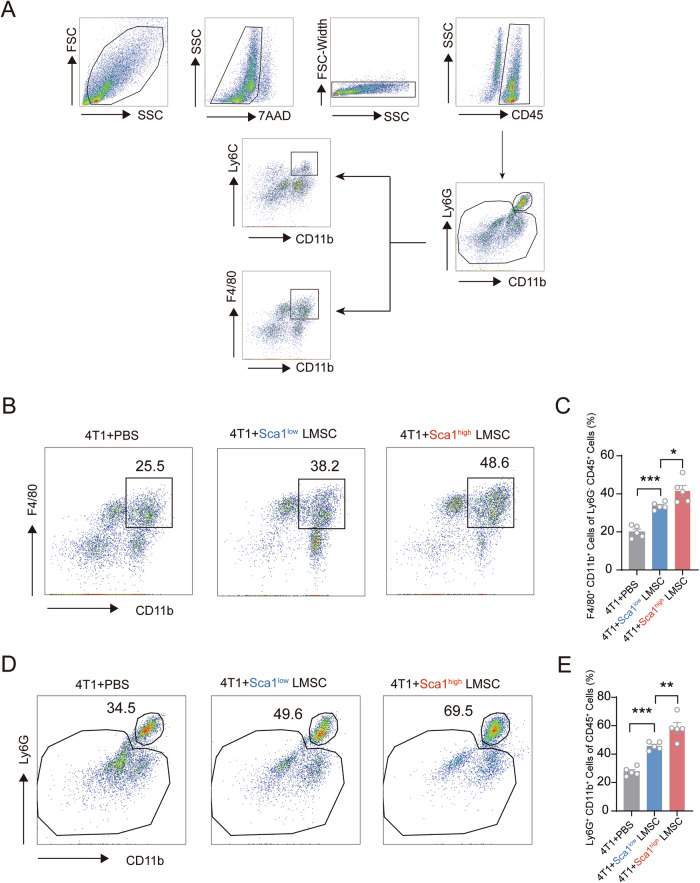
Co-injection of 4T1 and Sca-1^high^ LMSCs facilitate the recruitment of neutrophils and macrophages. **A** The immune cells in mice blood were analyzed by flow cytometry, the gating strategy of flow cytometry is shown. **B**, **C** CD11b^+^ F4/80^+^ cells were further increased after co-injection of 4T1 and Sca-1^high^ LMSCs compared with Sca-1^low^ LMSCs (*n* = 5). **D**, **E** CD11b^+^ Ly6G^+^ cells were also further elevated in tumor-bearing mice after injection of Sca-1^high^ LMSCs (*n* = 5). Data are mean ± SEM of biologically independent samples. Statistical analysis was determined by one-way ANOVA with Tukey’s multiple comparisons test. *p < 0.05, **p < 0.01, ***p < 0.001.

It has been well-established that immune cells play a crucial role in tumor metastasis [7, 10]. Building on our previous findings, which showed that Sca-1^high^ LMSCs exhibited high expression of CCL2 (a classical chemokine for macrophage recruitment) and CXCL1 (a ligand for CXCR2), and considering the known expression of CXCR2 in neutrophils [11], we investigated whether Sca-1^high^ LMSCs promote neutrophil accumulation in lung tissue. Using a defined gating strategy (Fig. 4A), we analyzed immune cell infiltration in the lung following tumor metastasis. Remarkably, we observed increased accumulation of CD11b^+^ F4/80^+^ cells in the lung tissue of mice co-injected with 4T1 cells and Sca-1^low^ LMSCs. Moreover, the administration of Sca-1^high^ LMSCs further augmented the recruitment of CD11b^+^ F4/80^+^ cells into the lung compared to Sca-1^low^ LMSCs (Fig. 4B, C). Additionally, the accumulation of CD11b^+^ Ly6G^+^ cells in the lungs was also elevated in mice injected with 4T1 cells and Sca-1^high^ LMSCs compared to those co-injected with Sca-1^low^ LMSCs (Fig. 4D, E). These findings suggest that Sca-1^high^ LMSCs are more potent in promoting neutrophil and macrophage infiltration into metastatic niches.

### Macrophage infiltration blockage abolishes the pro-metastatic effect of Sca-1^high^ LMSCs

**Fig. 5:**
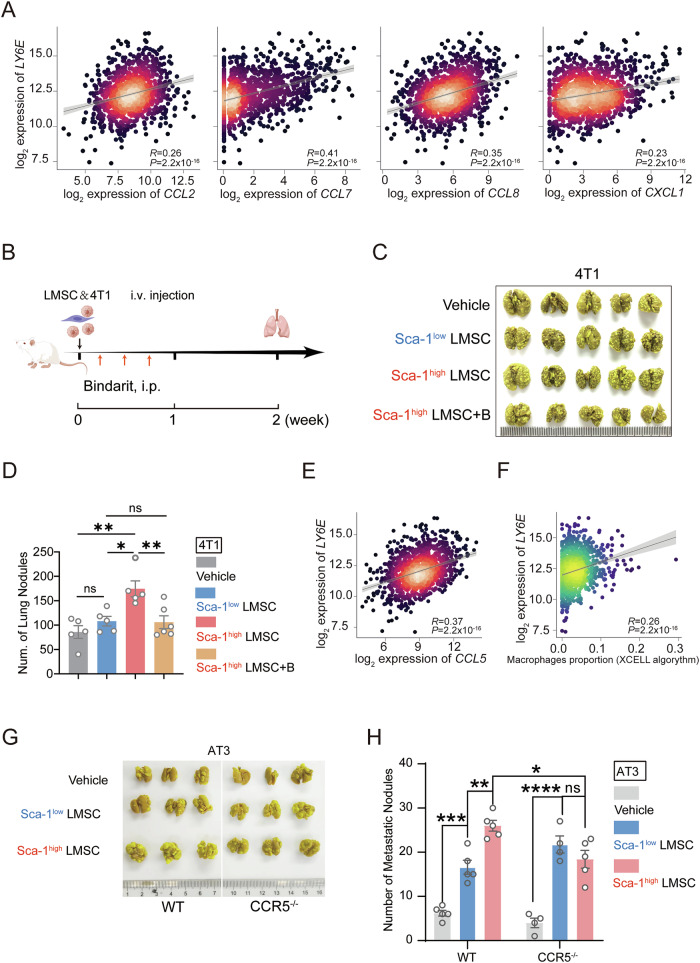
The blockade of macrophage infiltration abolished the tumor promotion potential of Sca-1^high^ LMSCs. **A** Correlation between *LY6E* and *CCL2/7/8* or *CXCL1* expression levels in breast cancer tissues from TCGA BRCA. **B** Schematic illustration of blockade of macrophages infiltration after co-injection of 4T1 and LMSCs. **C**, **D** Lung nodules in mice treated by tumor cells and LMSCs were stained by Bouin’s solution (*n* = 5 or 6). **E** Correlation between *LY6E* and *CCl5* expression levels in breast cancer tissues from TCGA BRCA. **F** Correlation between *LY6E* expression and predicted proportion of macrophages in breast cancer tissues from TCGA BRCA using xCell algorithm. **G**, **H** Wild type or Ccr5^−/−^ mice were treated by AT-3 and LMSCs injection (*n* = 4 or 5). The colonization of AT-3 cells in lungs was analyzed by Bouin’s staining. Data are mean ± SEM of biologically independent samples. Statistical analysis was determined by one-way ANOVA with Tukey’s multiple comparisons test (**D**), two-way ANOVA with Tukey’s multiple comparisons test (**H**). *p < 0.05, **p < 0.01, ***p < 0.001, ****p < 0.0001.

CCR2 and CCR5 are highly expressed in macrophages, which can be recruited by chemokines such as CCL2, CCL5, and CCL7. To investigate the correlation between CCL2/7/8 levels and Sca-1 expression levels, we analyzed TCGA BRCA data and saw a positive correlation between *CCL2/7/8* or *CXCL1* expression level and *LY6E* expression in cancer tissues (Fig. 5A). To further explore the critical role of macrophages in mediating the pro-metastasis effects of Sca-1^high^ LMSCs, we used Bindarit to inhibit the production of monocyte chemotactic proteins CCL2/7/8, thereby blocking the macrophage recruitment. After co-injecting 4T1 cells with Sca-1^high^ LMSCs, mice were administered Bindarit (10 mg/kg) intraperitoneally 3 times (Fig. 5B) and sacrificed 2 weeks later. Notably, Sca-1^high^ LMSC-driven lung metastasis was significantly reduced following Bindarit intervention (Fig. 5C). Moreover, following Bindarit treatment, Sca-1^high^ LMSCs were less effective in promoting lung colonization (Fig. 5D). Further analysis of the TCGA BRCA data revealed a positive correlation between *CCL5* and *LY6E* level in cancer tissues (Fig. 5E). Furthermore, we observed a positive correlation between LY6E expression and predicted proportion of macrophages in TCGA BRCA tumors by using xCell deconvolution algorithm [12] (Fig. 5F). To further confirm a critical role of macrophages in mediating the metastasis-promoting effect of Sca-1^high^ LMSCs, CCR5-deficient (Ccr5^−/−^) mice were employed. Since CCR5 is predominantly expressed on monocyte/macrophages, its deficiency inhibits macrophage chemotaxis. As expected, Ccr5^−/−^ mice exhibited reduced lung nodule formation compared to WT mice. More importantly, Sca-1^high^ LMSCs failed to promoting tumor growth when co-injected with AT-3 cells in Ccr5^−/−^ mice (Fig. 5G, H). Collectively, these results demonstrate that macrophages play a pivotal role in mediating the metastasis-promoting effect of Sca-1^high^ LMSCs.

## Discussion

**Fig. 6:**
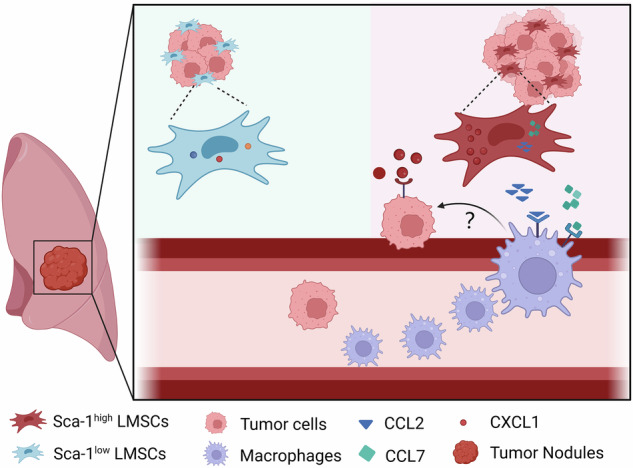
Schematic graph of pro-metastatic effect of Sca1^high^ LMSCs. Sca1^high^ LMSCs facilitate lung metastasis of breast tumor cells through the secretion of CXCL1 and CCL2/7. Compared with Sca1^low^ LMSCs, Sca1^high^ LMSCs exhibit elevated levels of CXCL1, attracting breast tumor cells directly. Meanwhile, CCL2/7 are also highly expressed in Sca1^high^ LMSCs, thus recruiting more macrophages into the lung tissue. Diagram created with BioRender.com.

MSCs exhibit high heterogeneity in their developmental lineages, stages of differentiation, and functional specializations. Tumor-associated MSCs likely consist of functionally distinct subsets. Our study shows that Sca-1 expression is higher in LMSCs at the metastatic stage compared to those in the pre-metastatic niche and is positively correlated with the expression of chemokines, including CCL2, CCL7, and CXCL1, that recruit myeloid cells. Consistently, macrophages were increasingly accumulated in lung tissue following co-injection of Sca-1^high^ LMSCs with 4T1 cells. In addition, CXCL1 directly mediated the migration of 4T1 cells. Thus, Sca-1^high^ MSCs promote breast cancer lung metastasis by recruiting both macrophages and cancer cells (Fig. 6). These findings provide valuable insight into the complex interplay between LMSCs, chemokines, immune cells, and tumor cells in the metastatic cascade of breast cancer to the lungs.

Tumors have often been described as ‘wounds that never heal’, as tumorigenesis is closely linked to chronic inflammation. This inflammatory microenvironment drives the recruitment of various responsive cell types, including macrophages, myeloid-derived suppressor cells, and MSCs [13, 14]. MSCs have been implicated in promoting tumor growth and metastasis across multiple cancer types [15–22]. Importantly, a growing body of research highlights the heterogeneous nature of MSCs [23–25]. Indeed, we recently demonstrated functional differences among LMSCs at various stages of breast cancer development [7]. Notably, metastatic LMSCs exhibited upregulated complement C3 expression when stimulated with Th2 cytokines, which led to increased neutrophil recruitment to pre-metastatic sites, promoting tumor metastasis. Consistent with these findings, our study also revealed elevated expression of Sca-1 along with the progression of breast cancer lung metastasis. Considering that Sca-1^high^ LMSCs promote lung metastasis of breast cancer by recruiting myeloid cells, which generally facilitate tumor colonization and growth, Sca-1^high^ LMSCs may also promote tumor metastasis to other organs. Further investigation is warranted to this possibility and to elucidate the key factors that drive Sca-1 expression in LMSCs.

Sca-1 expression is highly dynamic across various tissues, and its proper regulation is essential for tissue development and homeostasis. Despite its recognized importance, the biology of Sca-1 remains partially understood. Sca-1 functions as a co-regulator in lipid raft signaling, influencing stem cell fate [26] and acting as a cell-surface signaling protein and adhesion molecule [27]. It also plays a role in cell differentiation and signaling, modulating key pathways such as TGF-β [28, 29], ERK, and PI3K [30]. Sca-1 deficiency has been linked to increased fibrosis during tissue regeneration due to reduced MMP activity and impaired ECM turnover [31, 32]. In our study, we observed significant heterogeneity between Sca-1^high^ LMSCs and Sca-1^low^ LMSCs. Sca-1^high^ LMSCs exhibited markedly higher levels of cytokines, including CCL2, CCL7, and CXCL1, compared to Sca-1^low^ LMSCs. The Sca-1^high^ LMSCs promote metastasis both directly and indirectly through the secretion of cytokines. These findings suggest that Sca-1 plays a critical role in regulating the secretion of various cytokines. However, the exact mechanism behind this remains elusive.

The chemokine CCL2 acts on the macrophage receptor CCR2, and studies in mice with deletions in CCL2 or CCR2 have shown that macrophage infiltration and the conditioning lesion response in dorsal root ganglia are impaired (FRG) [33]. Our data indicate that tumors with high *LY6E* expression tend to have higher levels of macrophages. Moreover, in vitro, Bindrait effectively inhibited the pro-metastatic effects of Sca-1^high^ LMSCs, indicating that macrophages mediate the pro-metastatic effect.

Metastasis is a key determinant of cancer prognosis, and understanding how the pre-metastatic niche (PMN) or tumor micro-environment (TME) is established has garnered significant interest. Given the critical role of MSCs in the PMN and TME, targeting Sca-1^high^ MSCs in the TME has emerged as a potential strategy to improve outcomes for cancer patients. By targeting Sca-1^high^ LMSCs in human breast cancer, it may be possible to disrupt the establishment of the PMN in the lungs and inhibit metastatic progression.

## Methods and materials

### Animals

Female BALB/c mice and C57BL6/J mice were purchased from GemPharmatech. *Ccr5*^−/−^ mice (B6;129P2-Ccr5tm1Kuz/J) were purchased from Jackson Lab. All animals were maintained under specific pathogen-free conditions and were 6–8 weeks old for our experiments. The mice were randomly grouped according to a random number table. 5 × 10^5^ 4T1 or AT-3 and 1 × 10^5^ LMSCs were co-injected into the mice intravenously. Two weeks after cell administration, the mice were euthanized, and tumor nodules in the lungs were examined. The investigators were blinded to the group allocation during outcome assessment.

### Cell lines

Murine 4T1 and AT-3, mammary tumor cells, were cultured in DMEM/high medium containing 10% fetal bovine serum (FBS), 1% penicillin-streptomycin (Gibco, USA). All cells were recently authenticated by STR profiling and regularly tested to ensure they were mycoplasma-free.

### Lung-derived MSCs (LMSCs) isolation and culture

To isolate LMSCs, 6–8-week-old Balb/c mice were anesthetized. The whole lung was dissected and minced with a scalpel in DMEM/low medium. The resulting single cell and lung tissue suspension was plate in a 10-cm^2^ cell culture dish at a density of a whole lung/dish. These cells were cultured in complete DMEM/low medium consisting of 10% fetal bovine serum (FBS), 2 mmol/L-glutamine, and 1% penicillin and streptomycin (Gibco, USA). After 24 h, nonadherent cells were removed by 2 washes with phosphate-buffered saline (PBS), and adherent cells were cultured until they reached confluence. Cells were then trypsinized (0.25% trypsin with 0.1% EDTA) and sub-cultured at a density of 3 × 10^5^ cells/10 cm^2^ dish.

### Phenotype analysis of LMSCs

Cultured LMSCs were harvested, and flow cytometry was performed to analyse the phenotype after Sca-1 micromagnetic bead purification. Cells were washed with PBS, and then incubated with Sca-1 (eBioscience, 45-5981-82), CD11b (BD, 557657), Ly6G (Invitrogen, 63-9668-82), F4/80 (Invitrogen, 25-4801-82), CD45 (Biolegend, 147712) antibodies for 30 min in PBS containing 5% FBS at 4 °C. Analysis was performed using FlowJo software.

### Western blotting analysis

Cells were lysed in RIPA buffer (Beyotime, China) containing 1 mM phenylmethanesulfonyl fluoride (PMSF, Beyotime) for 30 min on ice. Proteins were incubated overnight at 4 °C with primary antibodies against Sca-1 (abcam, ab255604), β-actin (CST, Proteintech, 60008-1-Ig). The blotting membranes were then incubated with anti-rabbit or anti-mouse secondary antibodies for 1 h at room temperature. Chemiluminescent reagents (NCM Biotech, China) were used to develop the blotting membranes. The images were captured by ImageQuant 800 (Cytiva, USA).

### RNA sequencing of LMSCs

LMSCs isolated from MMTV-PyMT mice at different tumor stages (*n* = 3 for each stage) were subjected to RNA-seq analysis. The accession number for RNA sequencing data deposited in NCBI Gene Expression Omnibus is GEO: GSE125591.

### Quantitative real-time PCR

Total RNA was isolated using RNAfast200 Kit (Fastagen Biotech, Shanghai, China). Reverse transcription PCR was finished using the PrimeScriptTM RT Master Kit (TakaRa Biotech, Beijing, China). The mRNA expression was quantified by Real-Time PCR with SYBR Green Master Mix (ThermoFisher Scientific, USA). The relative abundance of mRNA was compared with endogenous β-actin mRNA. The mouse primer sequences were as follows: *Ccl2*-F: TTAAAAACCTGGATCGGAACCAA; *Ccl2*-R: GCATTAGCTTCAGATTTACGGGT; *Ccl3*-F: TTCTCTGTACCATGACACTCTGC; *Ccl3*-R: CGTGGAATCTTCCGGCTGTAG; *Ccl4*-F: TTCCTGCTGTTTCTCTTACACCT; *Ccl4*-R: CTGTCTGCCTCTTTTGGTCAG; *Ccl5*-F: GCTGCTTTGCCTACCTCTCC; *Ccl5*-R: TCGAGTGACAAACACGACTGC; *Ccl6*-F: GCTGGCCTCATACAAGAAATGG; *Ccl6*-R: GCTTAGGCACCTCTGAACTCTC; *Ccl7*-F: ATCACCAGTAGTCGGTGTCCCT; *Ccl7*-R: TCCATGCCCTTCTTTGTCTTG; *Ccl11*-F: GAATCACCAACAACAGATGCAC; *Ccl11*-R: ATCCTGGACCCACTTCTTCTT; *Ccl17*-F: TACCATGAGGTCACTTCAGATGC; *Ccl17*-R: GCACTCTCGGCCTACATTGG; *Ccl19*-F: CCTGGGAACATCGTGAAAGC; *Ccl19*-R: TAGTGTGGTGAACACAACAGC; *Ccl20*-F: GCCTCTCGTACATACAGACGC; *Ccl20*-R: CCAGTTCTGCTTTGGATCAGC; *Ccl22*-F: CTCTGCCATCACGTTTAGTGAA; *Ccl22*-R: GACGGTTATCAAAACAACGCC; *Ccl24*-F: TCTTGCTGCACGTCCTTTATT; *Ccl24*-R: GCATCCAGTTTTTGTATGTGCC; *Ccl25*-F: TTACCAGCACAGGATCAAATGG; *Ccl25*-R: CGGAAGTAGAATCTCACAGCAC; *Ccl26*-F: TTCTTCGATTTGGGTCTCCTTG; *Ccl26*-R: GTGCAGCTCTTGTCGGTGAA; *Ccl27*-F: AGGAGGATTGTCCACATGGAA; *Ccl27*-R: CTTGGCGTTCTAACCACCGA; *Ccl28*-F: GATGAGAGCCTCAGAGGTAAAGA; *Ccl28*-R: CTTTCTCGTAGTGTGCCCTTTT; *Cxcl1*-F: ACTGCACCCAAACCGAAGTC; *Cxcl1*-R: TGGGGACACCTTTTAGCATCTT; *Cxcl2*-F: CCAACCACCAGGCTACAGG; *Cxcl2*-R: GCGTCACACTCAAGCTCTG; *Cxcl9*-F: TCCTTTTGGGCATCATCTTCC; *Cxcl9*-R: TTTGTAGTGGATCGTGCCTCG; *Cxcl10*-F: CCAAGTGCTGCCGTCATTTTC; *Cxcl10*-R: GGCTCGCAGGGATGATTTCAA; *Cxcl11*-F: GGCTTCCTTATGTTCAAACAGGG; *Cxcl11*-F: GCCGTTACTCGGGTAAATTACA; *Cxcl12*-F: TGCATCAGTGACGGTAAACCA; *Cxcl12*-R: TTCTTCAGCCGTGCAACAATC; *Ccr1*-F: TACTCTGGAAACACAGACTCACT; *Ccr1*-R: ACAGCAGTCTTTTGGCATGG; *Ccr2*-F: AGAACTGTAGATCTTTGGGGAAACT; *Ccr2*-R: GTCTTTGCAGGCAGCTGAAC; *Ccr3*-F: TCGAGCCCGAACTGTGACT; *Ccr3*-R: CCTCTGGATAGCGAGGACTG; *Ccr4*-F: GGAAGGTATCAAGGCATTTGGG; *Ccr4*-R: GTACACGTCCGTCATGGACTT; *Ccr5*-F: TTTTCAAGGGTCAGTTCCGAC; *Ccr5*-R: GGAAGACCATCATGTTACCCAC; *Ccr6*-F: TGTACGAGTCGGTGTGCTTC; *Ccr6*-R: GGTAGGTATCCGTCATGGTCTTG; *Cxcr1*-F: TCTGGACTAATCCTGAGGGTG; *Cxcr1*-R: GCCTGTTGGTTATTGGAACTCTC; *Cxcr2*-F: ATGCCCTCTATTCTGCCAGAT; *Cxcr2*-R: GTGCTCCGGTTGTATAAGATGAC; *Cxcr3*-F: TACCTTGAGGTTAGTGAACGTCA; *Cxcr3*-R: CGCTCTCGTTTTCCCCATAATC; *Cxcr4*-F: CTTCTGGGCAGTTGATGCCAT; *Cxcr4*-R: CTGTTGGTGGCGTGGACAAT.

### Flow cytometry

Cells were washed with PBS and then were resuspended in fluorescence-activated cell sorting (FACS) buffer (PBS with 5% FBS) at a concentration of 5 × 10^5^ cells per 50 μL. These single-cell suspensions were incubated at 4 °C for 25 min with specific antibodies as described. FACS analysis was performed with FlowJo software.

### Trans-well assay

For trans-well migration and invasion assay, 5 × 10^5^ cells cultured in 400 μL medium with 10% FBS were plated in the upper chamber of a non-coated trans-well insert. In the lower chamber, 600 μL medium with 50% Sca-1^high^ or Sca-1^low^ LMSCs culture supernatant, respectively, was used as a chemo-attractant to encourage cell migration. Cells were incubated for 24 h, and those cells that did not migrate or invade were removed using a cotton swab. All cells were stained using crystal violet staining and counted under a light microscope. We selected five random views to count the target cells, and each experiment was repeated independently three times.

### Quantify of breast cancer metastasis nodules

Mice were sacrificed, and the lungs were surgically removed, followed by staining with Bouin’s solution. The lung metastasis was determined by counting the tumor nodules on the lungs.

### Bioinformatic analyses

The TCGA BRCA gene expression values were retrieved using Xena Browser (https://xenabrowser.net/). The differential expression analysis was performed using DESeq2 package from Xena Browser. Genes with *P* < 0.05 and abs(log2FC)>1 were considered significantly enriched. The values were represented as volcano plot using ggplot2 package. Correlation between *LY6E* and other genes’ expression levels was visualized using ggpointdensity and viridis packages. Pathway enrichment analysis was carried out using fgsea package. xCell predicted proportion of macrophages in TCGA BRCA samples was obtained from Timer2.o website (http://timer.cistrome.org/).

### Statistical analysis

Data were shown as mean ± SEM. Each result was confirmed by two or three repeated experiments. The significance was determined by a two-tailed unpaired Student *t*-test or one-way ANOVA with multiple comparisons as appropriate with Prism software (GraphPad9). *p < 0.05, **p < 0.01, ***p < 0.001, ****p < 0.0001. All of the data met the assumptions of the tests. The exact statistical parameters were presented in figure legends.

## Supplementary information

Supplementary Material

## Data Availability

All original data are available from the authors under request. TCGA BRCA data were obtained using UCSC Xena Browser (https://xenabrowser.net/).
