# Supplementary figures and images for: Mesenchymal stromal cells highly expressing Sca-1 promote breast cancer lung metastasis through recruiting myeloid cells
Source: Cell Death Dis. 2025 Jul 9;16(1):507. doi: 10.1038/s41419-025-07845-0 (PMC12241558; doi:10.1038/s41419-025-07845-0)

**Figure 1F**


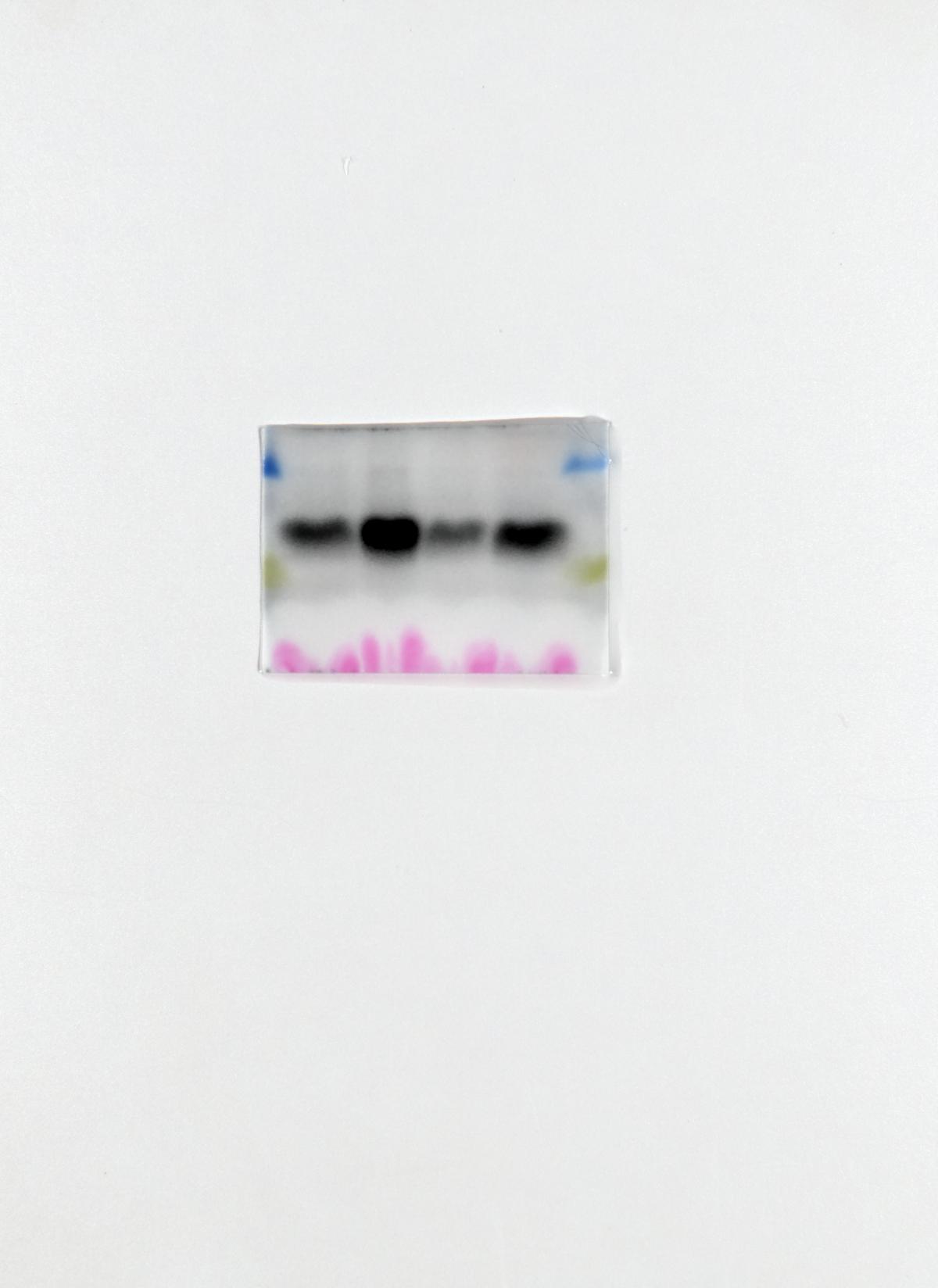


**25 KD**

**Sca-1**

**14 KD**


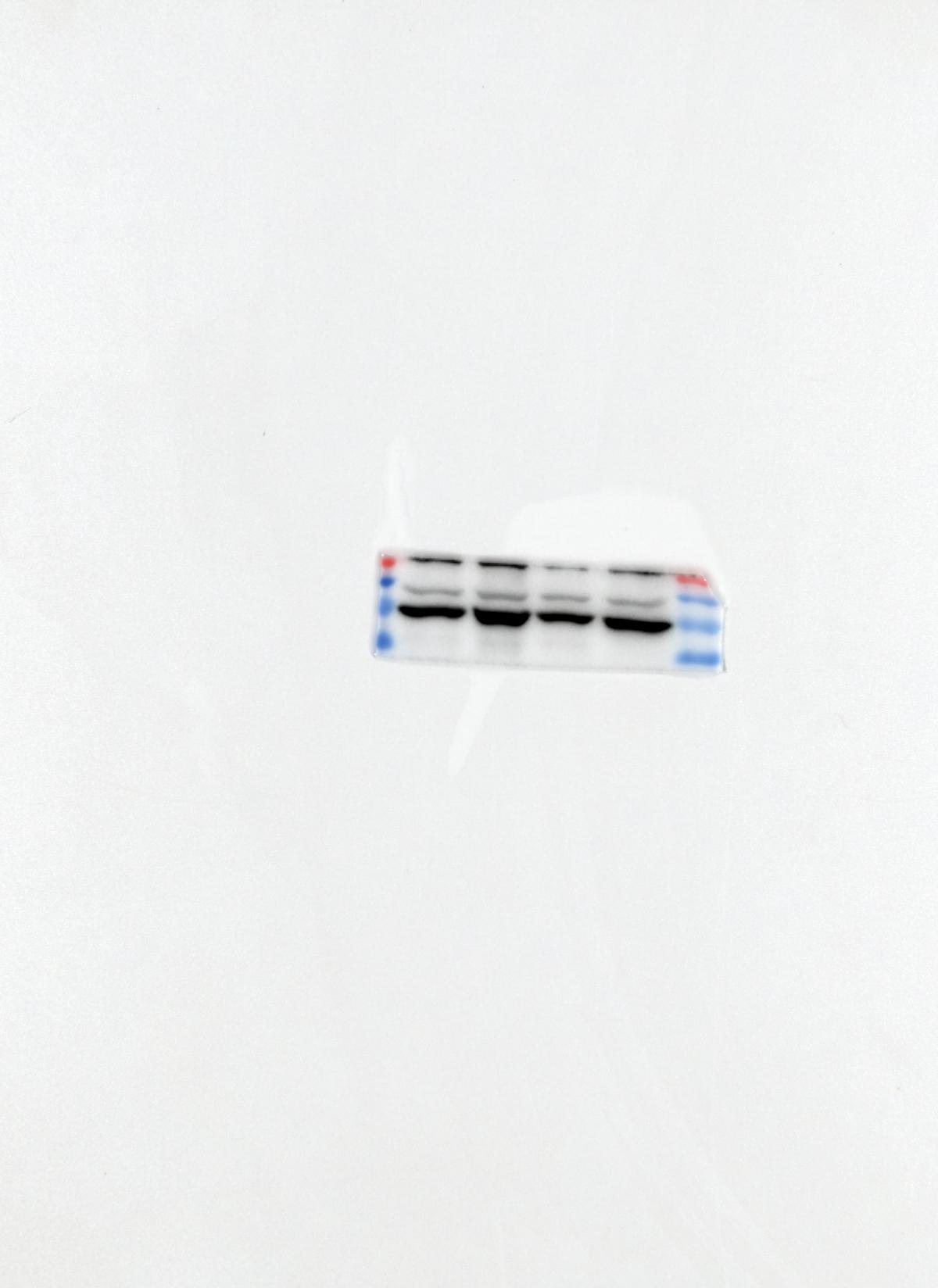


**53 KD**

**β-actin**

**43 KD**

**35 KD**

**Figure 1G**


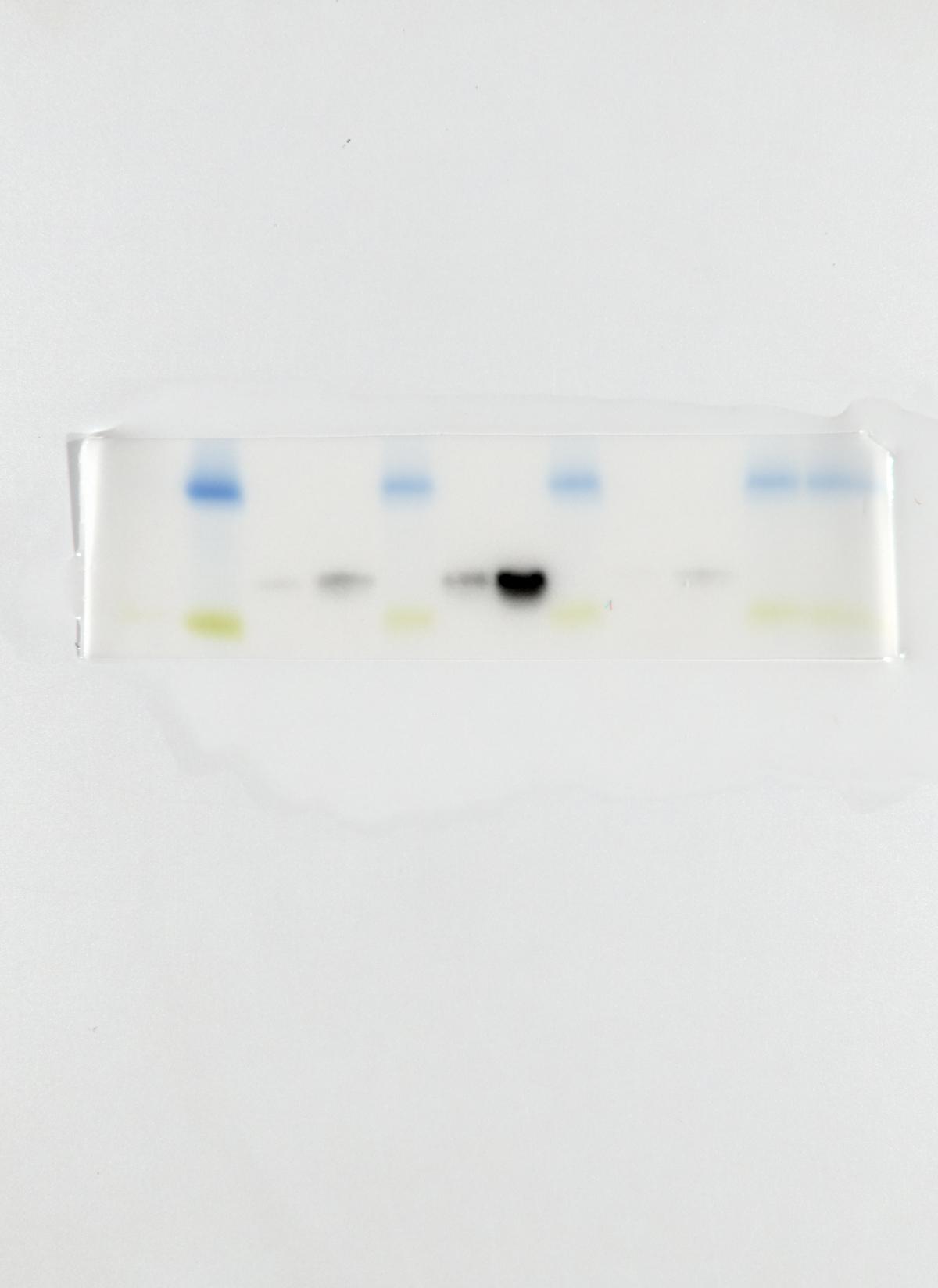


**25 KD**

**Sca-1**

**14 KD**


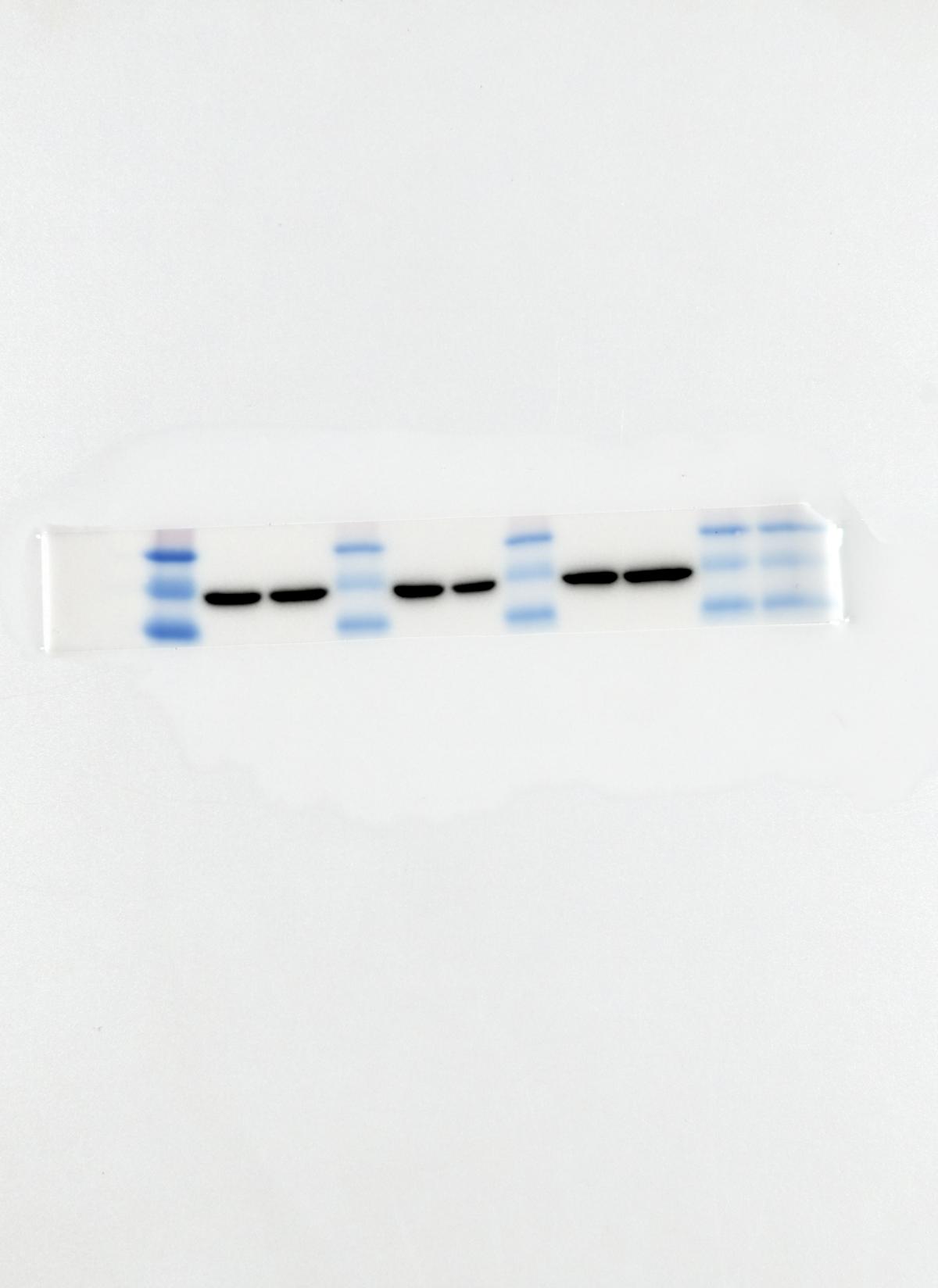


**53 KD**

**43 KD**

**35 KD**

**β-actin**

**Figure 1H**


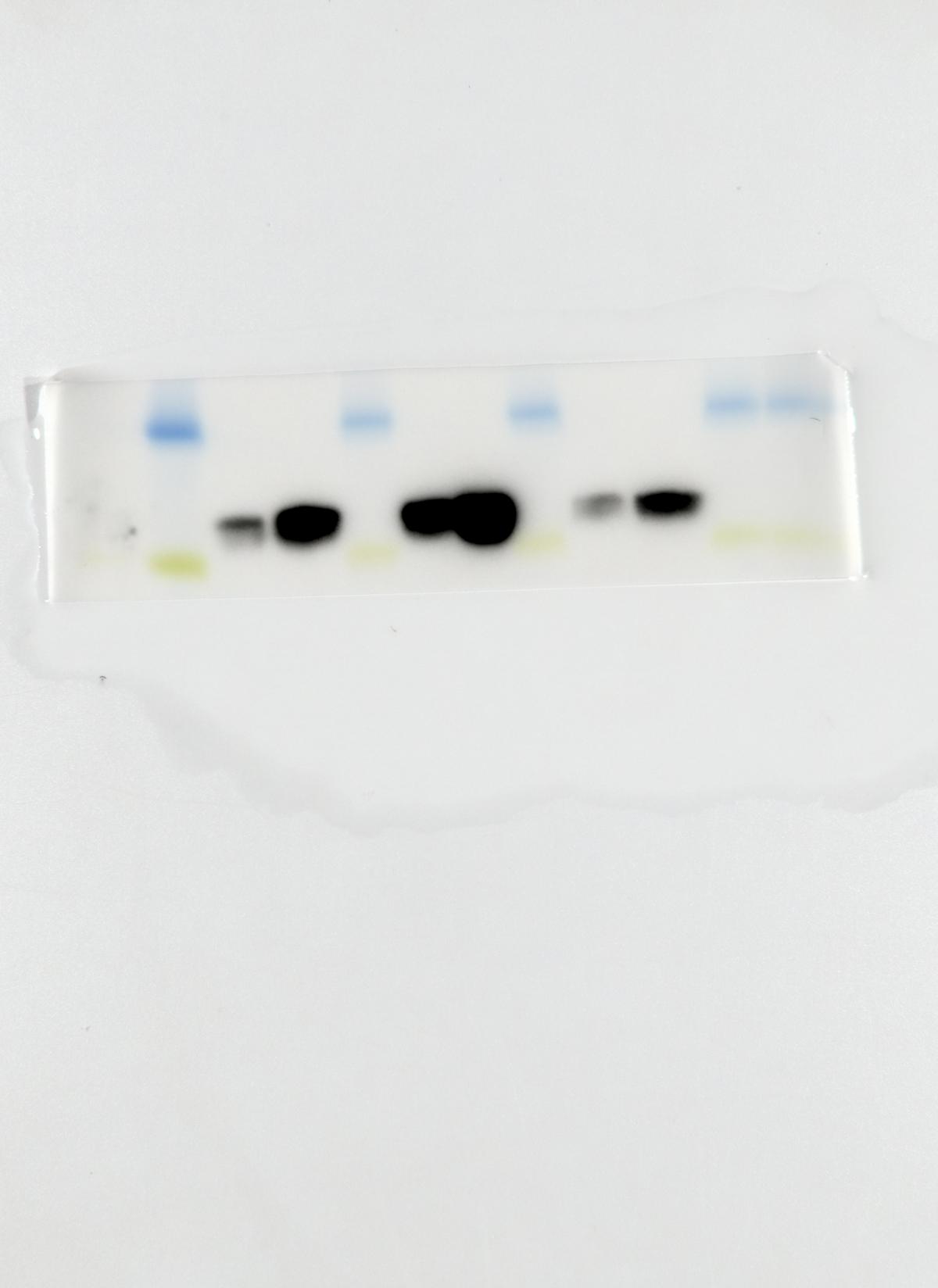


**25 KD**

**Sca-1**

**14 KD**


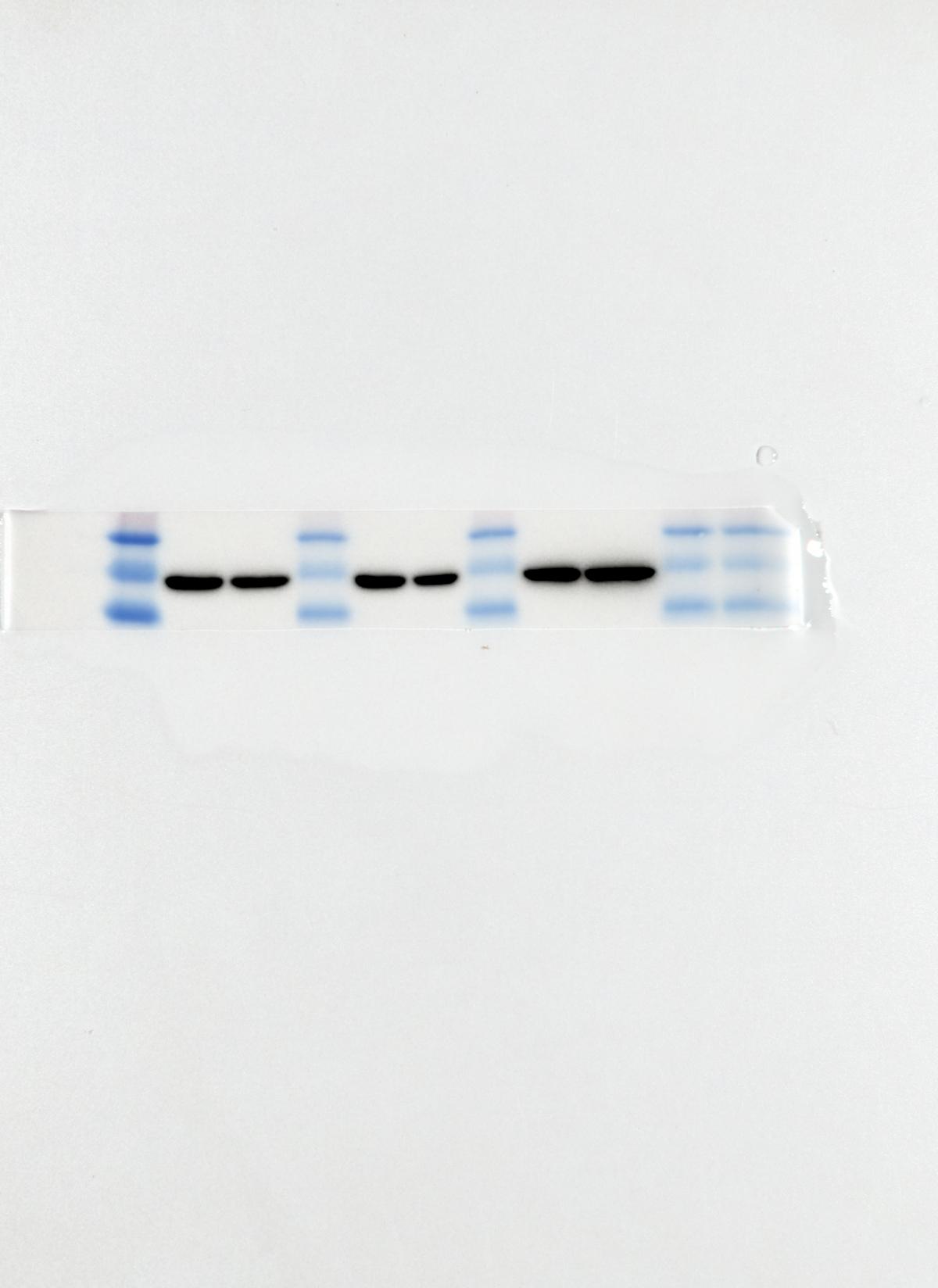


**β-actin**

**53 KD**

**43 KD**

**35 KD**

Supplement: Supplementary file 1 — Supplementary Material [file 41419_2025_7845_MOESM1_ESM.docx]
